# Threshold Dynamics of a Stochastic *SIR* Model with Vertical Transmission and Vaccination

**DOI:** 10.1155/2017/4820183

**Published:** 2017-07-06

**Authors:** Anqi Miao, Jian Zhang, Tongqian Zhang, B. G. Sampath Aruna Pradeep

**Affiliations:** ^1^College of Mathematics and Systems Science, Shandong University of Science and Technology, Qingdao 266590, China; ^2^State Key Laboratory of Mining Disaster Prevention and Control Co-Founded by Shandong Province and the Ministry of Science and Technology, Shandong University of Science and Technology, Qingdao 266590, China; ^3^Department of Mathematics, University of Ruhuna, 81000 Matara, Sri Lanka

## Abstract

A stochastic *SIR* model with vertical transmission and vaccination is proposed and investigated in this paper. The threshold dynamics are explored when the noise is small. The conditions for the extinction or persistence of infectious diseases are deduced. Our results show that large noise can lead to the extinction of infectious diseases which is conducive to epidemic diseases control.

## 1. Introduction

**Figure 1:**
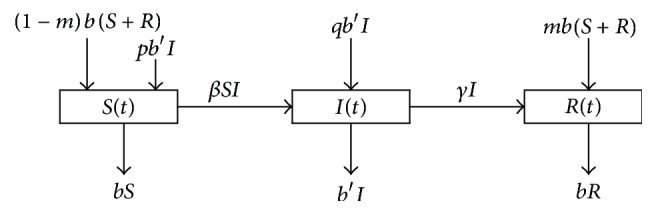
The compartmental diagram for the *SIR* model with vertical transmission and vaccination.

The history of mankind is filled with struggle with diseases. Infectious diseases such as smallpox, cholera, plague of leprosy, diphtheria, syphilis, typhus fever, malaria, rabies, and tuberculosis have threatened the health of human beings. People have realized the importance of quantitative studies on the spread of infectious diseases to predict and to control them. It can be known from referring to the literature [1–4] that, with the aid of the establishment of infectious disease models, people can understand the crucial laws of infectious diseases and provide reliable and enough information to predict and control infectious diseases. For example, as early as 1760, Bernoulli and Blower [5] proposed the first mathematical model in epidemiology for studying the spread and inoculation of smallpox. Further, in 1927, Kermack and McKendrick [6] proposed the concept of the so-called “compartmental model,” in which all the population was classified into three compartments: susceptible compartment *S*, infected compartment *I*, and removed compartment *R*. It is assumed in the model that the susceptible class can transform into the infective class through contact with infected individuals, and the infectives can recover through treatment so that they have permanent immunity. Therefore, it is now well known that many scholars have paid attention to *SIR* models; as a result, it can be seen in the literature that a large number of mathematical models of ordinary differential equations, delay differential equations, and partial differential equations have been constructed to study the spread of infectious diseases (see, e.g., [7–23]). In the last decades, we observed that scholars published few papers in scientific journals related to mathematics considering infectious diseases with vertical transmission which are transmitted from parents to their offspring (e.g., [1, 24–26]). Although scholars neglect the effect of vertical transmission, it is very important to study the real situation of the transmission of infectious diseases. The current diseases affecting humanity such as AIDS [27–31], Chagas' disease [32–34], hepatitis B [35, 36], and hepatitis C [37] are vertically transmitted. From this, it can be clearly seen that mathematical modeling including vertical transmission, horizontal transmission, and vaccination [38, 39] is more realistic than without them. Therefore, in this study, we have focused our attention on this and an *SIR* epidemic model involving vertical transmission and vaccination was proposed as follows [1, 24] (see Figure 1):

(1) S˙t=−βStIt+1−mbSt+Rt+pb′It−bSt,I˙t=βStIt+qb′It−b′It−γIt,R˙t=γIt−bRt+mbSt+Rt,

where *S*(*t*), *I*(*t*), and *R*(*t*) represent the members of the susceptible, the infectious, and the removed or the recovered members from infection, respectively. *b* is the birth and death rate of *S*(*t*) and *R*(*t*), *b*′ is the birth and death rate of *I*(*t*), *β* is the contact rate, and *m*  (0 < *m* < 1) is the vaccination proportion to the newborn from *S*(*t*) and *R*(*t*). Then, constant *p*  (0 < *p* < 1) is the proportion of the offspring of infective parents that are susceptible individuals and *p* + *q* = 1.   *γ* is the recovery rate of the infective individuals. Obviously, the total population size is normalized to one, and the basic reproductive number of system (1) is *R*_0_ = *β*(1 − *m*)/(*pb*′ + *γ*). By constructing a Lyapunov function and using the LaSalle invariance principle, we can show that if *R*_0_ < 1, the infection-free equilibrium *P*_0_(1 − *m*, 0, *m*) is globally asymptotically stable, while if *R*_0_ > 1, the infection-free equilibrium *P*_0_ is unstable and the endemic equilibrium *P*^*∗*^(*S*^*∗*^, *I*^*∗*^, *R*^*∗*^) is globally asymptotically stable.

In fact, the spread of diseases is inevitably disturbed by the influence of random factors; the stochastic epidemic system is more in line with the actual situation. Therefore, epidemic systems described by stochastic differential equations have been paid extensive attention in recent years (see, e.g., [40–46]). Various stochastic perturbation approaches have been introduced into epidemic systems and excellent results have been obtained. In this study, our main objective is to introduce four approaches. The first one is to analyze epidemic systems including the environment noise by using the method of time Markov chain (see, e.g., [47–51]). The second one is to consider the parameters' perturbation (see, e.g., [52–72]). The third one is to introduce Lévy jump noise into the system [73–75]. The fourth one is to investigate stochastic perturbation around the positive equilibria of deterministic systems (see, e.g., [41, 42, 76–78]).

Parameter perturbation induced by white noises is an important and common form to describe the effect of stochasticity. In this paper, we adopt the perturbation with white noises, that is, $\beta \to \beta +\sigma \dot{B}(t)$, where *B*(*t*) is a standard Brownian motion with intensity *σ*^2^ > 0. Then, the resultant system transforms into the following form:

(2) dSt=−βStIt+1−mbSt+Rt+pb′It−bStdt−σStItdBt,dIt=βStIt+qb′It−b′It−γItdt+σStItdBt,dRt=γIt−bRt+mbSt+Rtdt.

This paper is organized as follows. In Section 3, we will discuss the extinction of infectious diseases and explore the conditions leading to the extinction of infectious diseases. In Section 4, we will deduce the condition for a disease in order to be persistent.

## 2. Preliminaries

Throughout this paper, we let *ℝ*^*d*^: be the *d*-dimensional Euclidean space. *ℝ*_+_^*d*^≔{*x* ∈ *ℝ*^*d*^ : *x*_*i*_ > 0, 1 ≤ *i* ≤ *d*}, that is, the positive cone.

Let {*B*_*t*_}_*t*≥0_ be a one-dimensional Brownian motion defined on the complete probability space (*Ω*, *ℱ*, *𝒫*) adapted to the filtration {*ℱ*}_*t*≥0_. Let *ℒ*^1^(*ℝ*_+_; *ℝ*^*d*^) denote the family of all *ℝ*^*d*^-valued measurable {*ℱ*_*t*_}-adapted processes *f* = {*f*(*t*)}_*t*≥0_ such that

(3) $$\begin{array}{c} \overset{T}{\underset{0}{\int }}\left|\;f\left(\;t\;\right)\;\right|dt<\infty \;a.s.\text{ for every }T>0. \end{array}$$

Let *C*^2,1^(*ℝ*^*d*^ × *ℝ*_+_; *ℝ*) denote the family of all real-valued functions *V*(*x*, *t*) defined on *ℝ*^*d*^ × *ℝ*_+_ such that they are continuously twice differentiable in *x* and once in *t*. We set

(4) Vt=∂V∂t,Vx=∂V∂x1,∂V∂x2,…,∂V∂xd,Vxx=∂2V∂xi∂xjd×d=∂2V∂x1∂x1⋯∂2V∂x1∂xd⋮⋮∂2V∂xd∂x1⋯∂2V∂xd∂xd.

Clearly, when *V* ∈ *C*^2,1^(*R* × *R*_+_; *R*), we have *V*_*x*_ = ∂*V*/∂*x*, *V*_*xx*_ = ∂^2^*V*/∂*x*^2^. Then, we have the following.

**Lemma 1 (one-dimensional Itô's formula [40, 79, 80]).**

Let *x*(*t*) be an Itô process on *t* ≥ 0 with the stochastic differential

(5) $$\begin{array}{c} dx\left(\;t\;\right)=f\left(\;t\;\right)dt+g\left(\;t\;\right)dB_{t}, \end{array}$$

where *f* ∈ *ℒ*^1^(*ℝ*_+_; *ℝ*) and *g* ∈ *ℒ*^2^(*ℝ*_+_; *ℝ*). Let *V* ∈ *C*^2,1^(*ℝ*^*d*^ × *ℝ*_+_; *ℝ*). Then, *V*(*x*(*t*), *t*) is again an Itô process with the stochastic differential given by

(6) dVxt,t=Vtxt,t+Vxxt,tft+12Vxxxt,tg2tdt+Vxxt,t·gtdBt,

almost surely.

By using the methods from Lahrouz and Omari [81], we can prove the following lemma.

**Lemma 2 .**

For any initial value (*S*(0), *I*(0), *R*(0)) ∈ *R*_+_^3^, there exists a unique solution (*S*(*t*), *I*(*t*), *R*(*t*)) to system (2) on *t* ≥ 0, and the solution will remain in *R*_+_^3^ with probability one, namely.

**Lemma 3 .**

On the basis of Lemma 2, if *S*(0) + *I*(0) + *R*(0) ≤ 1, then *S*(*t*) + *I*(*t*) + *R*(*t*) ≤ 1, almost surely. Thus, the region Γ = {(*S*, *I*, *R*) ∈ *R*_+_^3^ : *S* > 0, *I* ≥ 0, *R* > 0, *S* + *I* + *R* ≤ 1} is a positively invariant set of system (2).

## 3. Extinction

In this section, we deduce the condition which will cause a disease to die out.

**Definition 4 .**

For system (2), the infected individual *I*(*t*) is said to be extinctive if lim_*t*→+*∞*_*I*(*t*) = 0, almost surely.

Let us introduce

(7) $$\begin{array}{c} R^{*}=\frac{R_{0}}{1-m}-\frac{\sigma^{2}}{2\left(pb^{\prime }+\gamma \right)} \end{array}$$

for convenience; then, we have the following results that we have mentioned in the following theorem.

**Theorem 5 .**

If *σ*^2^ > max⁡{*β*, *β*^2^/2(*pb*′ + *γ*)} or *σ*^2^ < *β* and *R*^*∗*^ < 1, then the infected individual of system (2) goes to extinction almost surely.

**Proof**

Let (*S*(*t*), *I*(*t*), *R*(*t*)) be a solution of system (2) with initial value (*S*(0), *I*(0), *R*(0)) ∈ *R*_+_^3^. Applying Itô's formula to the second equation of system (2) leads to

(8) dln⁡It=βSt−pb′+γ−σ22S2tdt+σStdBt.

Integrating both sides of (8) from 0 to *t* gives

(9) ln⁡It=∫0tβSτ−σ22S2τdτ−pb′+γt+Mt+ln⁡I0,

where *M*(*t*) = ∫_0_^*t*^*σS*(*τ*)d*B*(*τ*) and *M*(*t*) is the local continuous martingale with *M*(0) = 0. Next, we have two cases to be discussed, depending on whether *σ*^2^ > *β*.

If *σ*^2^ > *β*, we can easily see from (9) that

(10) $$\begin{array}{c} \ln I\left(\;t\;\right)\leq \left(\;\frac{\beta^{2}}{2\sigma^{2}}-\left(\;pb^{\prime }+\gamma \;\right)\;\right)t+M\left(\;t\;\right)+\ln I\left(\;0\;\right). \end{array}$$

Dividing both sides of (10) by *t* > 0, we have

(11) $$\begin{array}{c} \frac{\ln I\left(t\right)}{t}\leq -\left(\;pb^{\prime }+\gamma -\frac{\beta^{2}}{2\sigma^{2}}\;\right)+\frac{M\left(t\right)}{t}+\frac{\ln I\left(0\right)}{t}. \end{array}$$

Since lim sup_*t*→*∞*_(〈*M*(*t*), *M*(*t*)〉_*t*_/*t*) < *σ*^2^ < *∞* almost surely, by the large number theorem for martingales (see, e.g., [53]), one can obtain that

(12) $$\begin{array}{c} \underset{t\to +\infty }{lim}\frac{M\left(t\right)}{t}=0. \end{array}$$

Then, taking the limit superior on both sides of (11) leads to

(13) $$\begin{array}{c} \underset{t\to +\infty }{lim\;sup}\frac{\ln I\left(t\right)}{t}\leq -\left(\;pb^{\prime }+\gamma -\frac{\beta^{2}}{2\sigma^{2}}\;\right)<0, \end{array}$$

when *σ*^2^ > *β*^2^/2(*pb*′ + *γ*), which implies lim_*t*→+*∞*_*I*(*t*) = 0.

If *σ*^2^ < *β*, similarly, one can have that

(14) $$\begin{array}{c} \ln I\left(\;t\;\right)\leq \left(\;\beta -\left(\;pb^{\prime }+\gamma \;\right)-\frac{\sigma^{2}}{2}\;\right)t+M\left(\;t\;\right)+\ln I\left(\;0\;\right). \end{array}$$

Dividing both sides of (14) by *t* > 0, we have

(15) ln⁡Itt≤pb′+γβpb′+γ−σ22pb′+γ−1+Mtt+ln⁡I0t.

By taking the superior limit on both sides of (15), one can have that

(16) $$\begin{array}{c} \underset{t\to +\infty }{lim\;sup}\frac{\ln I\left(t\right)}{t}\leq \left(\;pb^{\prime }+\gamma \;\right)\left(\;R^{*}-1\;\right). \end{array}$$

Then, when *R*^*∗*^ < 1, we obtain

(17) $$\begin{array}{c} \underset{t\to +\infty }{lim\;sup}\frac{\ln I\left(t\right)}{t}<0, \end{array}$$

which implies lim_*t*→+*∞*_*I*(*t*) = 0. This completes the proof of Theorem 5.

**Remark 6 .**

Theorem 5 shows that when *σ*^2^ > max⁡{*β*, *β*^2^/2(*pb*′ + *γ*)}, the infectious disease of system (2) goes to extinction almost surely; namely, large white noise stochastic disturbance is conducive to control infectious diseases. When the white noise is not large and *R*^*∗*^ < 1, the infectious disease of system (2) also goes to extinction almost surely; then, *R*^*∗*^ is the threshold associated with the extinction of infectious diseases.

## 4. Persistence in Mean

**Definition 7 .**

For system (2), the infected individual *I*(*t*) is said to be permanent in mean if lim inf_*t*→+*∞*_〈*I*(*t*)〉>0, almost surely, where 〈*I*(*t*)〉 is defined as (1/*t*)∫_0_^*t*^*I*(*τ*)d*τ*.

Let us denote

(18) $$\begin{array}{c} R^{**}=R_{0}-\frac{\sigma^{2}}{2\left(pb^{\prime }+\gamma \right)} \end{array}$$

for convenience; then, we have the following results that we have mentioned in the following theorem.

**Theorem 8 .**

If *ℛ*^*∗∗*^ > 1, then the infected individual *I*(*t*) is persistent in mean; moreover, *I*(*t*) satisfies

(19) $$\begin{array}{c} \underset{t\to +\infty }{lim\;inf}\langle \;I\left(\;t\;\right)\;\rangle \geq \frac{\left(pb^{\prime }+\gamma \right)}{\beta \left(1-m+\gamma /b\right)}\left(\;R^{**}-1\;\right), \end{array}$$

almost surely.

**Proof**

Integrating from 0 to *t* and dividing by *t*  (*t* > 0) on both sides of the third equation of system (2) yield

(20) Rt−R0tγIt+mbSt−1−mbRt≜Θt.

Note that 〈*S*(*t*)〉+〈*I*(*t*)〉+〈*R*(*t*)〉 = 1; then, one can get

(21) $$\begin{array}{c} \langle \;S\left(\;t\;\right)\;\rangle =\left(\;1-m\;\right)+\frac{\Theta \left(t\right)}{b}-\left(\;1-m+\frac{\gamma }{b}\;\right)\langle \;I\left(\;t\;\right)\;\rangle . \end{array}$$

Applying Itô's formula gives

(22) dln⁡ItβSt−pb′+γ−σ22S2tdt+σStdBt≥βSt−pb′+γ−σ22dt+σStdBt.

Integrating from 0 to *t* and dividing by *t*  (*t* > 0) on both sides of (22) yield

(23) ln⁡It−ln⁡I0t≥βSt−pb′+γ+σ22+Mtt=β1−m+Θtb−1−m+γbIt−pb′+γ+σ22+Mtt.

From (23), we obtain

(24) It≥1β1−m+γ/bβ1−m−pb′+γ−σ22+1β1−m+γ/bβΘtb−ln⁡It−ln⁡I0t+Mtt.

Since both *I*(*t*) ≤ 1 and *R*(*t*) ≤ 1, then one has lim_*t*→+*∞*_(*R*(*t*)/*t*) = 0, lim_*t*→+*∞*_(ln⁡*I*(*t*)/*t*) = 0, and lim_*t*→+*∞*_Θ(*t*) = 0. Note that lim_*t*→+*∞*_(*M*(*t*)/*t*) = 0; by taking the inferior limit of both sides of (24), we have

(25) lim inft→+∞It≥1β1−m+γ/bβ1−m−pb′−γ−σ22=pb′+γβ1−m+γ/bR∗∗−1.

This completes the proof of Theorem 8.

**Remark 9 .**

Theorems 5 and 8 show that the condition for the disease to die out or persist depends on the intensity of white noise disturbances strongly. And small white noise disturbances will be beneficial for long-term prevalence of the disease; conversely, large white noise disturbances may cause the epidemic disease to die out.

## 5. Conclusion and Numerical Simulation

In this paper, a stochastic *SIR* system with vertical transmission and vaccination is proposed. The threshold dynamics depending on the stochastic perturbation are deduced by using the theory of stochastic differential equation and inequality technique. Our results show that the dynamics of the stochastic system are different with the deterministic case due to the effect of stochastic perturbation, and the persistent diseases in the deterministic system may be eliminated under the stochastic perturbation.

In the following, by employing the Euler Maruyama (EM) method [40], we perform some numerical simulations to illustrate the extinction and persistence of the diseases in the stochastic system and corresponding deterministic system for comparison.

**Figure 2:**
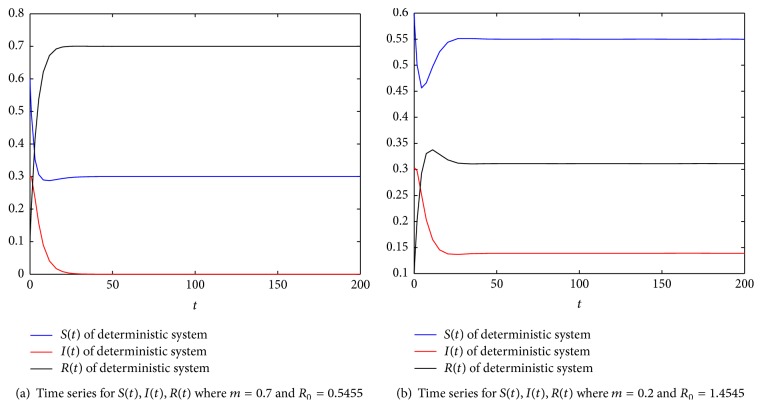
Illustration for the deterministic *SIR* system where *β* = 0.8, *p* = 0.6, *b* = 0.2, *b*′ = 0.4, and *γ* = 0.2.

For numerical simulations, we set parameters as *m* = 0.7, *β* = 0.8, *p* = 0.6, *b* = 0.2, *b*′ = 0.4, and *γ* = 0.2 in system (1). A simple computation shows that *R*_0_ = 0.5455 < 1, and then system (1) has a stable infection-free equilibrium *P*_0_(0.3,0, 0.7), which implies that the disease of system (1) will be eliminated ultimately (see Figure 2(a)). If we change *m* = 0.7 to *m* = 0.2, in this case, *R*_0_ = 1.4545 > 1, and then system (1) has a stable infection equilibrium *P*^*∗*^(0.55,0.3111,0.1389), which implies that the disease of system (1) will be persistent ultimately (see Figure 2(b)).

**Figure 3:**
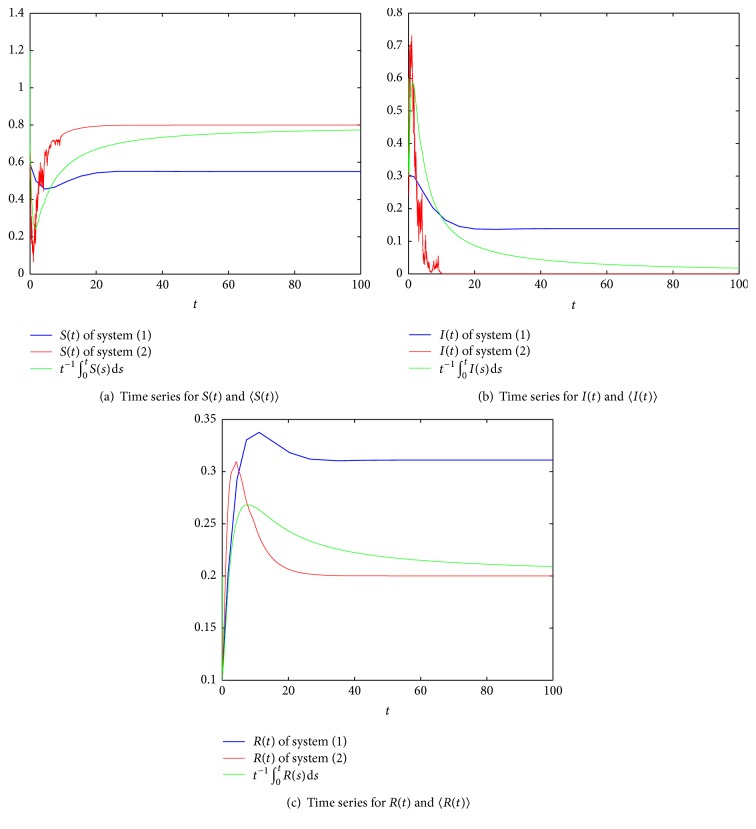
Comparison of the deterministic system and stochastic system, where *m* = 0.2, *β* = 0.8, *p* = 0.6, *b* = 0.2, *b*′ = 0.4, *γ* = 0.2, *σ* = 0.9, and *R*_0_ = 1.4545 > 1.

**Figure 4:**
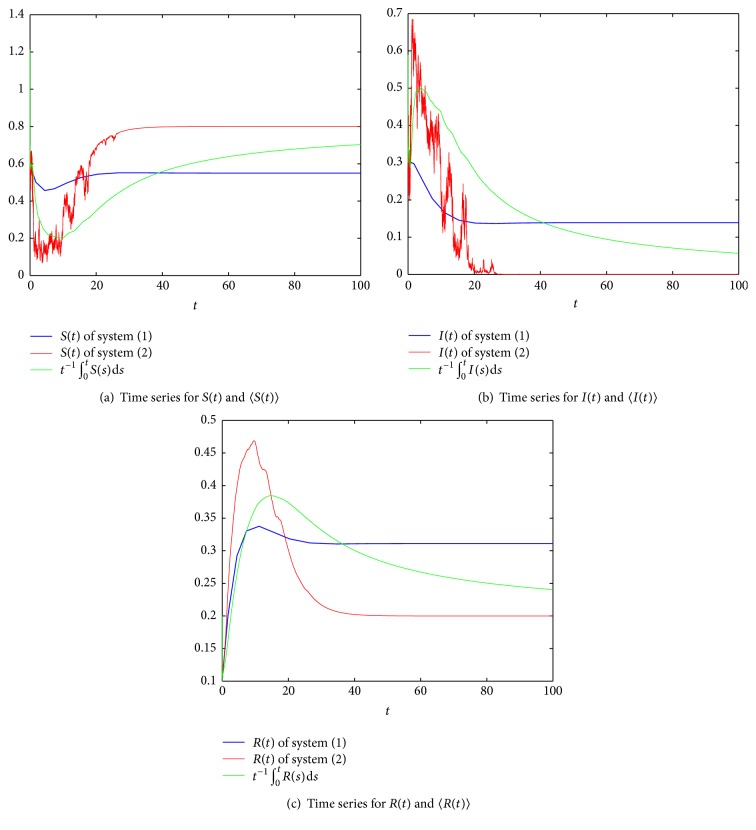
Comparison of the deterministic system and stochastic system, where *m* = 0.2, *β* = 0.8, *p* = 0.6, *b* = 0.2, *b*′ = 0.4, *γ* = 0.2, *σ* = 0.85, *R*^*∗*^ = 0.9972, and *R*_0_ = 1.4545 > 1.

**Figure 5:**
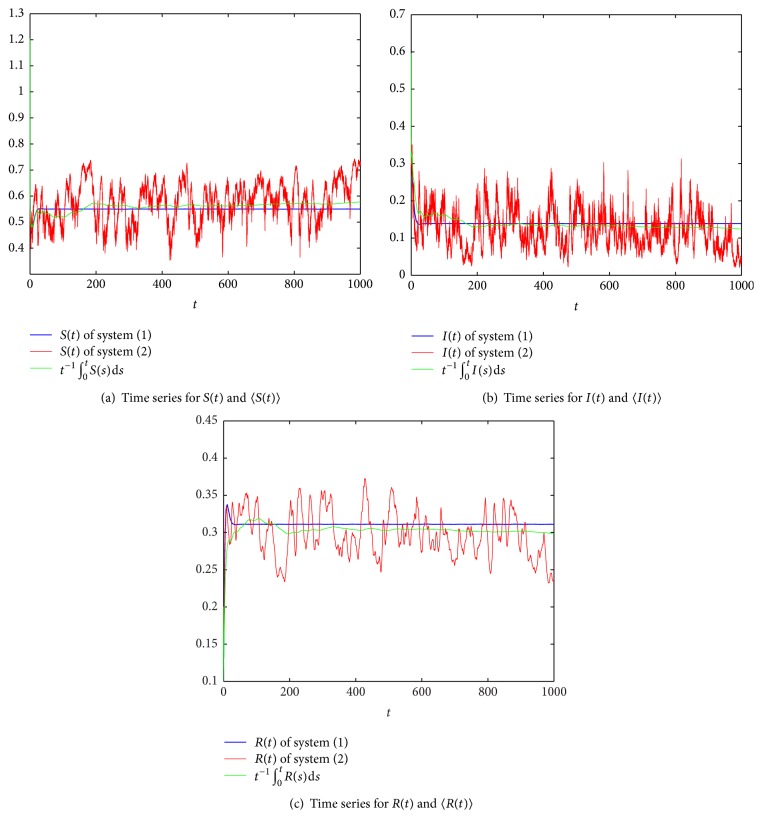
Comparison of the deterministic system and stochastic system, where *m* = 0.2, *β* = 0.8, *p* = 0.6, *b* = 0.2, *b*′ = 0.4, *γ* = 0.2, *σ* = 0.2, *R*^*∗∗*^ = 1.4091, and *R*_0_ = 1.4545 > 1.

Next, we consider the effect of stochastic white noise based on the persistent system. Let *σ* = 0.9, and obviously, *σ*^2^ > max⁡{*β*, *β*^2^/2(*pb*′ + *γ*)}; by Theorem 5, the disease dies out under a large white noise disturbance (see Figure 3). If we change *σ* to 0.85, in this case, *σ*^2^ < *β*^2^/2(*pb*′ + *γ*) and *R*^*∗*^ = 0.9972 = <1; then, by Theorem 5, the disease dies out (see Figure 4). If we reduce the intensity of noise *σ* to 0.2, obviously, *R*^*∗∗*^ = 1.4091 > 1; by Theorem 8, the disease is persistent (see Figure 5).
